# The Incidence and Risk Factors of Surgical Site Infection Following Cesarean Section

**DOI:** 10.1155/nrp/4980949

**Published:** 2025-05-28

**Authors:** Hind Ghannam Alruwaili, Wedad M. Almutairi, Areej A. Abunar

**Affiliations:** ^1^Obstetrics and Gynecology Nursing, King Saud University Medical City, Riyadh, Saudi Arabia; ^2^Obstetrics and Gynecology Nursing, King Abdulaziz University, Jeddah, Saudi Arabia; ^3^Maternity and Child Health Nursing, King Abdulaziz University, Jeddah, Saudi Arabia

**Keywords:** cesarean delivery, infection, risk factors, Sakaka, surgical site infection

## Abstract

**Background:** The incidence of surgical site infection (SSI) following cesarean section (CS) is between 3% and 15% worldwide. There is a paucity of evidence regarding the incidence and risk factors of SSI following CS in Saudi Arabia. Globally, infection is the third greatest cause of maternal deaths. There are many risk factors associated with SSI post-CS such as age, previous history of CS, medical diseases, and a high number of vaginal examinations.

**Methods:** A cross-sectional prospective descriptive study among women who underwent CS.

**Setting:** Gynecology and obstetrics clinics in a maternity and children's hospital.

**Sample:** A convenience sample of 124 mothers within 30 days after CS was used; data were collected from July 2021 to August 2021. This study found that the incidence of SSI after CS was 4% of the mothers who underwent CS operations in the Maternity and Children Hospital in Sakaka. Besides, the results showed that there is a significant association between the type of anesthesia (spinal) and SSI following CS (Chi = 4.288, *p* ≤ 0.05). To conclude that the incidence of SSI following CS was 4%, comparable to the international rate, and spinal anesthesia was the confirmed risk factor in our sample. Further studies should be carried out with larger samples and in more than one hospital in Sakaka, Saudi Arabia.

## 1. Introduction

The inclusion of surgery within the realm of public health is now widely acknowledged in developing nations. The measurement of operational treatment consistency in these environments is of utmost importance as program managers enhance their surgical capacity. Nevertheless, locating the requisite metrics poses a challenging task. Moreover, certain programs, characterized by significantly more stringent limitations, are exclusively designated for emergency situations. It is imperative to establish comprehensive programs that ensure the provision of high-quality care in advance [1].

According to the World Health Organization (WHO) [2], there is a global incidence of 211 maternal deaths per 100,000 live births annually. According to the WHO [2], infection ranks as the third most significant contributor to maternal mortality on a global scale. According to Al-Kadri [3], there is a correlation between the incidence of infections and the rising prevalence of cesarean section (CS) deliveries. According to Nagy and Papp [4], there has been a notable rise in the overall proportion of cesarean deliveries over the course of the past decade, reaching nearly 21% of all births. Furthermore, it has been observed that this percentage continues to grow steadily each year, with an annual increase of approximately 4% in Saudi Arabia. According to a study conducted at King Abdulaziz Medical City [5], the prevalence of CS in Saudi Arabia in 2016 surpassed the maximum recommended rate of 15% set by the WHO, reaching a total of 27% of all deliveries. The global prevalence of surgical site infections (SSIs) after CS ranges from 3% to 15% according to a meta-analysis study conducted by Saeed et al. [6]. The prevalence of SSIs and SSI subsequent to CS in Saudi Arabia remains undetermined; however, a study conducted in Riyadh reported an SSI rate of 1.7% [7]. According to Gadeer et al. [8], the prevalence of SSIs subsequent to CS at King Abdulaziz Medical City in Jeddah City was recorded at 3.4%. From a physiological standpoint, the occurrence of infection subsequent to CS has significant ramifications. This includes imposing additional strain on the new mother and impeding the crucial process of mother–infant bonding and breastfeeding due to the necessity of separating the mother and infant [9]. SSIs have been found to have a negative impact on both the physical and psychological well-being of patients. Additionally, patients may also experience intangible costs, such as increased pain and anxiety. Furthermore, the extended absence of a patient from their residence and workplace can also result in distress for both the patient and their family members. Consequently, research has demonstrated that extended periods of hospitalization and heightened morbidity resulting from the acquisition of a SSI have a detrimental effect on the health-related quality of life of patients [10]. Numerous studies have documented that patients who experience SSIs necessitate extended periods of hospitalization, reoperation, and readmission. Furthermore, these SSIs have been found to contribute to an elevated mortality rate. This is primarily due to the significant financial burden associated with SSIs, which includes additional costs related to medical personnel, diagnostic procedures, and therapeutic interventions [10]. Furthermore, the nation is confronted with economic obstacles as a consequence of the COVID-19 pandemic. The intricate nature of the economic ramifications, including morbidity and mortality, and the subsequent surgical or medical interventions, such as antibiotic therapy, is frequently observed [11].

The risk factors can be classified into three primary categories, namely, host factors, pregnancy-related factors, and procedural factors. The initial category encompasses factors associated with the host, such as maternal age (advanced or young), obesity, place of residence (rural or urban), prior history of CS, recurrent pregnancy loss, and pregestational diabetes mellitus (DM). The second category encompasses various factors that are associated with the pregnancy itself. These factors include hypertension (HTN), gestational diabetes mellitus (GDM), twin pregnancy, preterm rupture of membranes, an increased number of vaginal exams, utilization of epidural anesthesia, extended waiting periods for vaginal delivery prior to CS, implementation of internal fetal monitoring, and the presence of chorioamnionitis. The third category pertains to the CS procedure, encompassing emergency CS, and abstention from prophylactic antibiotic usage, instances involving uterine rupture and subsequent removal, extended surgical duration, and requirement for a blood transfusion, according to a study conducted by Zuarez-Easton et al. [12]. This study aims to address the existing gap in the literature regarding the limited information available on the prevalence of SSIs following CS and the associated risk factors in healthcare settings in Saudi Arabia. This highlights the importance and relevance of our study. To the best of our current knowledge, there is a lack of research conducted in Saudi Arabia regarding the incidence of SSIs following CS and the associated risk factors among women residing in Sakaka. Nevertheless, the present study provides support for additional analyses pertaining to obstetrics and gynecology matters, thereby contributing to the augmentation of research publications within the region. Hence, the primary objective of this investigation is to ascertain the prevalence of SSIs among women who have undergone CS at the Maternity and Children Hospital in Sakaka, Saudi Arabia. Additionally, this study seeks to identify the risk factors associated with such infections.

## 2. Methodology

### 2.1. Research Design

The study's design is a cross-sectional prospective descriptive design. A cross-sectional study is defined as collecting study data at a single time point [13, 14].

### 2.2. Research Setting

The current study was conducted in the outpatient department of the Maternity and Children Hospital in Sakaka, which is situated in northern Saudi Arabia. It is the only hospital in the region that specializes in gynecological and children's care, and this hospital belongs to the Ministry of Health and receives patients that are Saudi and non-Saudi. Moreover, it has a 150-bed facility and 11 clinics dedicated to gynecology, prenatal, postnatal, infertility, and pediatric care; it provides one-day procedures to give medication and fluids during follow-ups if needed to patients, and it has a dressing clinic dedicated to women who have already undergone surgeries who require follow-up surgery incision to remove sutures.

### 2.3. Sample Size

In the current study, the total population size was estimated electronically using the sample size calculator on the target community's website. The sample size was calculated using the estimated population size (CS per year), with a 5% margin of error and 95% confidence level. The calculated sample size was 123 women for the study.

### 2.4. Research Tool

The study tool was adopted from Alamri's (2020) study. The questionnaire contained five sections starting from sociodemographic data which includes age, educational level for women, occupation, income of participant, type of residency, and nationality, and then the medical history which include HTN, DM, heart disease (HD), and anemia; hemoglobin (Hb) antenatal level (< 11 g/dL, ≥ 11 g/dL); and body mass index (BMI), as well as the obstetric history and intraoperative and postoperative history. SSI following CS measured by a questionnaire using a multiple-choice Likert scale for symptoms in the first 30 days post-CS and question included was if women had been diagnosed by physician of SSI or not. The classifications of general infection symptoms including fever and local symptoms (REEDA) such as redness, localized swelling (edema), ecchymosis, purulent drainage, and wound closure complications. Finally, there were questions about the use of herbs. These yes-or-no questions asked the participants about what type of herbs they use, and Cronbach's coefficient alpha for the study domain of self-care management behavior was 0.742, which is good. Also, the tool's general validity coefficient was 0.903.

The inclusion criteria were as follows: Women post-CS and the first 30 days after CS.

### 2.5. Data Collection

The data collection period for the current study was 2 months, from July 2021 to August 2021. To find participants, the researcher went to outpatient department, from 8 a.m. to 2 p.m. Then, before proceeding with the study, the researcher introduced himself to them and explained to the participants the purpose of the study, promised to guarantee the anonymity of the participants, and obtained subject written consent, which was voluntarily after receiving verbal consent, taking into account the individual's ethical stances. In addition, the researcher explained to them that even if they agreed to participate in the study, they could withdraw at any time if they wished.

### 2.6. Statistical Analysis

The program used to enter the data was Microsoft Office Excel Program, and for analyzing the data, the program used was SPSS (including descriptive and inferential analyzing). The description used for sociodemographic characteristics included frequencies and percentages. In addition, inferential statistics were used, such as Fisher's exact test and the Mann–Whitney test, which provided answers about questions in the current study. Fisher's exact test is used when the table contains cells with zero counts or when expected frequencies in some cells are less than 5. We did not use any AI software in our study.

### 2.7. Ethical Considerations

An official written approval from the Deanship of Postgraduate Studies in King Abdulaziz University was received to collect data (NREC serial no: Ref. no. 2M.71). Also, approval was obtained from the research ethics committee in the hospital. Submitted papers or grants that contain confidential information are protected under research ethics for publication; this includes personnel files, trade or military secrets, and patient files [15]. Moreover, the confidentiality of the digital data did not include names or other personal data, and the removal of all identifiers at the time of publication will be ensured.

## 3. Results

A total of 124 post-CS women were collected using convenience sampling methods from obstetrics and gynecology clinics; most of them were aging between 30 and 39 years old with the highest percentage of secondary and more education; among these participants, 78.2% live in urban areas, while 21.8% live in rural areas. Moreover, 89.5% of participants are Saudis, while 10.5% are non-Saudis; elective CS was chosen by 45 (36.3%), and emergency CS was chosen by 79 (63.7%). Of the 124 post-CS women, 119 (96%) stated that they had not been diagnosed with SSI after CS during the first 30 days post-CS, while only five (4%) women confirmed their diagnosis of SSI, and Table 1 shows the main distribution of sociodemographic data among participants in this study.

In relation to the provided medical history data, it is evident that a significant proportion of participants, specifically 99.2%, do not exhibit HTN. Similarly, a substantial majority of participants, amounting to 97.6%, do not present with DM. Furthermore, the data indicate that a majority of participants, approximately 96.8%, do not manifest symptoms of anemia. Furthermore, it was observed that Hb levels during the antenatal phase were above 11 g/dL for the majority (95.7%) of participants, whereas 40.3% of them had Hb levels below 11 g/dL. Moreover, a majority of the participants, specifically 53.2%, exhibit obesity, whereas 31.5% are categorized as overweight. Furthermore, it should be noted that all cases of SSI were devoid of HD or any form of anemia. Furthermore, it was observed that 60.0% of the cases exhibited an Hb level equal to or greater than 11 mg/dL, whereas 40.0% of the cases displayed an Hb level below 11 mg/dL.

In relation to the data concerning obstetric history, the findings indicate that 44.4% of the participants have a gravida of 2–4, whereas 38.7% have a gravida of five or more. Furthermore, it should be noted that the majority, specifically 53.2%, exhibit a parity ranging from 2 to 4 times, whereas a smaller proportion, 26.2%, possess a parity of 5 times or greater. Furthermore, it is noteworthy that 80.6% of the participants in the study exhibit a gestational age (GA) falling within the range of 37–42 weeks, whereas the remaining 19.4% of participants have a GA below 37 weeks. With respect to the participants' prevalence of pregnancy induced hypertension (PIH), it was found that 87.9% of them do not exhibit signs of the disease. Furthermore, it is worth noting that majority of participants, 96.8%, did not have GDM. Fifty percent of the participants refrained from participating in a vaginal examination, whereas 34.7% of the participants underwent a vaginal examination on five or fewer occasions. Sixty percent had emergency CS, and 50% did not have a vaginal examination because it was either rejected by women or the cases were very urgent. In addition, 42.7% of emergency (CS) had fetal distress. Moreover, none of the participants exhibit chorioamnionitis or any other form of infection (Table 2).

The frequency and percentage of SSI cases among participants and the distribution of the study participants according to their intraoperative and postoperative history are shown in the following Table 3.

Regarding the frequency and percentage of SSI following CS cases among participant's with regard to their intraoperative and postoperative history which were only five, the results revealed that 60.0% of SSI cases had elective surgery, and 60.0% of them had CS due to previous CS, while 40.0% had CS due to fetal distress. Regarding prophylaxis antibiotics, 60.0% of SSI cases had antibiotics for > 1 h, while 40.0% had it for ≤ 1 h before surgery. In addition, 60.0% of SSI cases had spinal anesthesia, and they did not receive a blood transfusion. Regarding skin incision, all cases of SSI have Pfannenstiel skin incision, and the procedure duration of surgery for all cases was ≤ 1 h. And the SSI following CS among participants is 4.0%.

The majority (96%) did diagnoses with SSI following CS. Moreover, 2.4% of participants who have SSI following CS were hospitalized, while 97.6% were not. Moreover, 4.0% of participants who have SSI following CS received antibiotics, while 96.0% did not as shown in the following Tables 4 and 5.

The association between SSI following CS and related factors is shown in the following Tables 6, 7, 8 that were illustrated by Fisher's exact test; to find no significant association between the variables and the occurrence of SSI after CS, there was no relationship between the risk factors (age, GA, parity, history of CS, obesity, anemia, HTN, and diabetes) in the question and the occurrence of SSI following CS (age [*p* value = 0.747], GA [*p* value = 0.970], parity [*p* value = 0.485], history of previous CS [*p* value = 0.599], obesity [*p* value = 0.509], anemia [*p* value = 0.677], HTN [*p* value = 0.837], and DM [*p* value = 0.719]). The association between the dependent variable (incidence of SSI following CS) and independent variables (PROM, emergency CS, preterm labor, type of anesthesia, and blood transfusion) was examined; there was a significant association between the type of anesthesia and the occurrence of SSI after CS. Participants receiving spinal anesthesia had a significantly higher risk of SSI than those receiving general anesthesia (type of CS [*p* value = 0.260], type of anesthesia [*p* value ≤ 0.038], status of membrane [*p* value = 0.451], GA [*p* value = 0.970], and blood transfusion [*p* value ≤ 0.070]), while there is no significant association between HTN and the occurrence of SSI (*p* ≤ 0.05). In addition, there is no significant association between DM and the occurrence of SSI (*p* ≤ 0.05). Moreover, there is no significant association between anemia and SSI occurrence (*p* ≤ 0.05). Also, there is no significant association between participants' Hb and the occurrence of SSI (*p* ≤ 0.05), and there is no significant association between participants' BMI and the occurrence of SSI (*p* ≤ 0.05); in addition, there is no significant association between the number of pregnancies (gravida) and the occurrence of SSI (*p* ≤ 0.05). Furthermore, there is no significant association between the number of deliveries (parity) and the occurrence of SSI (*p* ≤ 0.05). Moreover, there is no significant association between GA, vaginal examination, the status of the membrane, and the occurrence of SSI (*p* ≤ 0.05). In addition, there is no significant association between GDM and the occurrence of SSI (*p* ≤ 0.05), and there is no significant association between chorioamnionitis and other infections and the occurrence of SSI (*p* ≤ 0.05). Fisher's exact test is used when the table contains cells with zero counts or when expected frequencies in some cells are less than 5.

Also, about 60.0% of SSI participants were hospitalized, while 40.0% were not. Moreover, all SSI cases received antibiotics, and there is a significant association between the type of anesthesia and the occurrence of SSI (*p* ≤ 0.05). Participants who have undergone spinal anesthesia have significantly more risk of having SSI than participants who have undergone general anesthesia. Moreover, there is no significant association between intraoperative and postoperative history and SSI occurrence (*p* ≤ 0.05). Also, there is no significant association between indications for surgery and SSI occurrence (*p* ≤ 0.05). In addition, there is no significant association between prophylaxis antibiotics and occurrence of SSI (*p* ≤ 0.05). Furthermore, there is no significant association between the type of incision and SSI occurrence (*p* ≤ 0.05). Moreover, there is no significant association between blood transfusion, procedure duration, and SSI occurrence (*p* ≤ 0.05).

Regarding the association between SSI following CS and symptoms experienced during the first 30 days as shown in the following table, there is a significant difference in the median score of having fever in the first 30 days after CS for SSI occurrence among participants (*p* ≤ 0.05). Participants who do not have SSI have a higher median score of having fever than those with SSI. In addition, there is a significant difference in the median score of having redness for the occurrence of SSI among participants (*p* ≤ 0.05). Participants who do not have SSI have a higher median score of redness than those who have SSI, and there is a substantial median score of having edema for SSI occurrence among participants (*p* ≤ 0.05). Participants who do not have SSI have a higher median score of edema than those who have SSI. Moreover, there is a significant difference in the median score of having purulent drainage for SSI occurrence among participants (*p* ≤ 0.05). Participants who do not have SSI have a higher median score of having purulent drainage than those with SSI.

In addition, there is a significant difference in the median score of having wound closure complications for the occurrence of SSI among participants (*p* ≤ 0.05). Participants who do not have SSI have a higher median score of having wound closure complications than participants who have SSI. Moreover, there is no significant difference in the median score of having ecchymosis for SSI occurrence among participants (*p* ≤ 0.05) Table 9.

## 4. Discussion

SSIs: The present study found a 4% incidence of SSI after CS, which is congruent with Gadeer [8] in Jeddah, Saudi Arabia, who found 3.4%. Alamri (2021) found 3% SSI after CS. This study found less SSIs after CS than in the United States and Ethiopia. Moulton et al. [16] identified a 5.5% incidence rate at the US academic institution. Gelaw et al. [17] found 6.8% frequency in an Ethiopian public hospital. The current study found more SSIs after CS than earlier studies in the United Arab Emirates and Kuwait. Alnajjar and Alashker [18] found 1.4% incidence in the United Arab Emirates. Alfouzan et al. [19] reported a 2.1% incidence rate in Kuwait. In developing nations such as Ethiopia, SSIs exceed the 3% threshold due to lower healthcare accessibility and educational attainment. Lijaemiro et al. [20] found that 15% of Addis Ababa participants had SSIs after CS. At Debre Markos Referral Hospital in the Amhara region of northwest Ethiopia, the incidence rate was 25.4%. Ketema et al. [21] found a higher prevalence of the disorder than prior investigations. According to Moulton et al. [16], SSIs after CS were greater in the United States than in Saudi Arabia. The prevalence of SSI following CS in the United States of America is higher may be due to the sample size in the United States is higher than in Saudi Arabia; in addition, the nature of demographics varies between societies.

Risk factors for SSIs: The present study found no significant association between age and SSI after CS. Regmi [22] claimed that risk variables including age increase the risk of SSIs after CS. Lijaemiro [20] found a significant association between maternal age and the study variable, showing that SSI prevalence increased with younger mothers. Chu [1] observed several risk variables for SSIs, including younger age. Wloch [23] observed that younger women are more susceptible to SSIs. Saeed [24] found similar results. Wloch [23] found that under-20s have considerable independent risk factors for wound infection. Alnajjar [18] and this study both included age. Age did not affect SSIs after CS in either study. This study found no significant association between SSIs after CS and medical history factors, possibly due to the small sample size and the lack of comparability between the groups of women who had SSI and those who did not. HTN and DM did not affect SSIs after CS. Regmi [22] observed a positive link between HTN and DM and SSIs after CS. Ayala's [25] study also found a link between DM and HTN. Our research found no statistically significant relationship between HD and SSI after cardiac surgery. Regmi [22] found that HD increases SSI risk. However, Moulton [16] and our investigation found no significant connection between HD and SSI after CS. Anemia does not cause SSIs after CS, according to the study. Jasim [26] found the same. Anemia was a major risk factor in Regmi's [22] study. Anemia increased the risk of CS SSIs. Puerperal sepsis and anemia are often seen together. In Ali's study [27], anemia was linked to SSIs after CS deliveries. Our research indicates that prenatal Hb levels do not predict SSIs after CS. Rose [28] found that normal Hb levels reduce the risk of SSIs. Karaca [29] found that preoperative Hb levels increased the risk of SSI in adolescent mothers. Farret [30] found no link between prenatal Hb levels and SSIs after CS. The present investigation found no significant association between BMI and SSI after CS. Shree [31] found the same. Vallegoe [32] found a positive link between greater BMI and SSIs after CS. In Ousey et al.'s study [33], only the BMI was associated with SSI. This also matches Moulton et al.'s study [16]. DM, HTN, anemia, HD, prenatal Hb levels, BMI, and the result of interest were not associated in this study.

These findings may be due to incomparable study groups, sample size, and homogeneity issues, a shortage of SSI cases after CS, and the presence of obese or overweight participants. Obstetric history factors, such as parity, GA, and gravida did not correlate with SSI after CS. However, Seligman [34] found a link between gravida and SSIs. The factor did not affect SSIs after CS. Vellejo [32] and Dessu [35] found no link between gravida and SSI after CS. Parity did not affect SSIs after CS. Contrary to Gomaa et al.'s study [36], parity was associated with SSI after CS. Chu [1] and Jido and Garba [37] observed no significant association between parity and SSI following CS. GDM was not associated with SSI after CS in our study. Regmi [22] and Vallego [32] found a link between GDM and SSI after CS, contrary to our findings. Shree [31] found that GDM was not associated with SSI after CS, supporting our findings. Our study found no significant link between PIH and postcesarean SSIs. Regime [22] found a link between PIH and post-CS SSIs. Shree et al. [31] observed no significant connection between PIH and SSI in CS, supporting our findings. The present investigation found no significant correlation between GA and postcesarean SSIs. Moulton [16] and Gomaa [36] found opposite results. Saeed et al. [24] found no link between GA and postcesarean SSIs. Contrary to Pathak et al. [38] and Regmi et al.'s study [22], the present study found no statistical significance for vaginal examinations. Vallejo et al.'s [32] vaginal examination study matches ours. This study found no significant connection between membrane status and SSI risk. Chu et al. [1] found that membrane status did increase SSI risk after CS. Chu et al. [1] and Rose et al. [28] supported these findings. Rose [28] observed that intact membranes before surgery reduced SSIs. This study confirms the findings of Saeed et al. [24] that membrane statuses did not increase the risk of SSIs after CS. The present investigation found no statistical relevance between preoperative infections and postoperative SSIs. Lijaemiro [20] found that preoperative illnesses such as HIV did not increase SSI risk. Ketema [21] found a stronger link between HIV positivity and SSI following CS. Chorioamnionitis was not associated with SSIs after CS, consistent with Vallejo [32]. However, Dessu [35] and Ketcheson [39] found that chorioamnionitis is a risk factor for SSIs after CS. Parity, gravida, GDM, PIH, GA, vaginal examination, fetal membrane status, previous infections, and chorioamnionitis were not significantly associated with SSIs after CS. Due to the researcher's inability to recruit more SSI patients after CS, the study's sample size, homogeneity, and duration were limited. The type of CS, the indication for surgery, the administration of prophylactic antibiotics, the type of anesthesia, the need for blood transfusion, the method of skin incision, and the duration of the procedure did not affect SSIs after CS. This contradicts Alfouzan et al. [19], who found that women who did not get prophylactic antibiotics had a greater SSI rate than those who did. These findings show that inadequate prophylactic antibiotics increase SSI risk. Prophylactic antibiotics were not associated with SSIs after CS in this study. Lijaemiro et al. [20] found no link between preventive antibiotics and SSIs after CSs. Haidar et al. [40] also found that intrapartum antibiotics reduced SSI rates. Pathak [38] found that the improper use of prophylactic antibiotics increased SSIs by a factor of five. Blood transfusion and SSIs after CS are not linked, according to this study. Dessu et al. [35] also found comparable results. However, Jasim et al. [41] found a link between increased blood loss during surgery and postcesarean SSIs. Dessu [35] also found no connection between blood loss and SSIs after CS. Our study found a link between spinal anesthesia and postcesarean SSIs. Similar to Jasim et al. [41], spinal anesthesia is associated with SSIs after CS. Moulton et al. [16] discovered that the general anesthesia increases the risk of SSIs compared to a US academic study. Alemye et al. [42] found that general anesthesia was associated with SSIs in CSs. The classification of CSs did not correlate with SSIs after CS, agreeing with Lijaemiro et al. [20]. This contradicts Alfouzan et al.'s study [19], who found a link between CS type and SSI after the surgery. Haider et al. [40] also found a favorable association between CS type and SSI risk. This study found no connection between surgery indication and other risk variables. Shree et al. [31] found a link between SSI and CS surgery. Regmi et al. [22] found no correlation between surgery indication and SSIs after CS. In CS, skin incision is not associated with SSI.

## 5. Conclusion

The prevalence of SSIs following CS in Sakaka region in Saudi Arabia is 4%. The risk of SSI following CS is greater in women who had spinal anesthesia in the current study. Our study suggests replicating the study with larger sample size and different settings to support the generalizability of the study findings.

## Figures and Tables

**Table 1:** Distribution of the study participants according to their age, education, employment, and income (*n* = 124).

Variables	Frequency	Percentage (%)
Age groups		
< 30 years	39	31.4
30–39 years	74	59.7
≥ 40 years	11	8.9
Education		
Illiterate	3	2.4
Elementary	18	14.5
Secondary and college or more	103	83.1
Employment		
Student	4	3.2
Housewife	90	72.6
Employee	30	24.2
Income		
More than enough	4	3.2
Just enough	116	93.6
Not enough	4	3.2

**Table 2:** Frequency and percentage in SSI following CS cases about their obstetric history (*n* = 5).

Variables	Frequency	Percentage (%)	
Gravida	Once	0	0.0
	2–4 times	3	60.0
	≥ 5 times	2	40.0

Para	Once	0	0.0
	2–4 times	3	60.0
	≥ 5 times	2	40.0

PIH	Yes	0	0.0
	No	5	100.0

GDM	Yes	0	0.0
	No	5	100.0

GA	< 37 weeks	1	20.0
	37–42 weeks	4	80.0
	> 43 weeks	0	0.0

Vaginal exam	Yes > 5 times	0	0.0
	Yes 5 ≤ times	1	20.0
	No	4	80.0

Status of membrane	Ruptured > 12 h	0	0.0
	Ruptured ≤ hours	0	0.0
	Intact	5	100.0

Chorioamnionitis	Yes	0	0.0
	No	5	100.0

Other infection	Yes	0	0.0
	No	5	100.0

**Table 3:** Distribution of the study participants according to their intraoperative and postoperative history (*n* = 124).

Variables	Frequency	Percentage (%)
Intraoperative and postoperative history		
Elective	45	36.3
Emergency	79	63.7
Indication for surgery		
Previous CS	53	42.7
Malpresentation	15	12.1
Fetal distress	56	45.2
Prophylaxis antibiotics		
≤ 1 h prior to surgery	74	59.7
> 1 h prior to surgery	50	40.3
Type of anesthesia		
Spinal	110	88.7
General	14	11.3
Blood transfusion		
Yes	48	38.7
No	76	61.3
Skin incision		
Pfannenstiel	124	100.0
Procedure duration		
≤ 1 h	117	94.4
> 1 h	7	5.6

**Table 4:** Surgical site infection following CS (*n* = 124).

Variables	Frequency	Percentage (%)
Incidence of SSI (diagnosis of SSI)		
Yes	5	4.0
No	119	96.0
Hospitalization		
Yes	3	2.4
No	121	97.6
Receiving antibiotics		
Yes	5	4.0
No	119	96.0

**Table 5:** Symptoms experienced by study participants in the first 30 days (*n* = 5).

Symptoms	*N*	%
Fever	4	32.2
Redness	4	36.6
Edema	4	34.0
Ecchymosis	2	30.8
Purulent drainage	5	28.2
Wound closure complications	5	30.2

**Table 6:** Association between SSI following CS and participants' medical history.

Medical history		SSI	No SSI	Chi-square	*p* value
Age	< 30 years	2 (5.1)	37 (94.9)	0.583	0.747
	30–39 years	3 (4.1)	71 (95.9)		
	40 years or more	0 (0.0)	11 (100.0)		

HTN	Yes	0 (0.0)	1 (0.8)	0.042	0.837
	No	5 (100.0)	118 (99.2)		

DM	Yes	0 (0.0)	3 (2.5)	0.129	0.719
	No	5 (100.0)	116 (97.5)		

HD	Yes	0 (0.0)	0 (0.0)	≤ 0.001	≤ 0.001
	No	5 (100.0)	119 (100.0)		

Anemia	Yes	0 (0.0)	4 (3.4)	0.174	0.677
	No	5 (100.0)	115 (96.6)		

Hb antenatal	< 11 g/dL	2 (40.0)	48 (40.3)	≤ 0.001	0.988
	11 g/dL	3 (60.0)	71 (59.7)		

BMI	Underweight (< 18.50)	0 (0.0)	2 (1.7)	2.320	0.509
	Normal (18.50–24.99)	0 (0.0)	17 (14.3)		
	Overweight (≥ 25.00)	3 (60.0)	36 (30.3)		
	Obese (≥ 30.00)	2 (40.0)	64 (53.8)		

**Table 7:** Association between SSI following CS and participants' obstetric history.

Obstetric history		SSI	No SSI	Chi-square	*p* value
Gravida	Once	0 (0.0)	21 (17.6)	1.172	0.557
	2–4 times	3 (60.0)	52 (43.7)		
	≥ 5 times	2 (40.0)	46 (38.7)		

Para	Once	0 (0.00)	25 (21.0)	1.446	0.485
	2–4 times	3 (60.0)	63 (52.9)		
	≥ 5 times	2 (40.0)	31 (26.1)		

PIH	Yes	0 (0.0)	15 (12.6)	0.717	0.397
	No	5 (100.0)	104 (87.4)		

GDM	Yes	0 (20.0)	4 (3.4)	0.174	0.677
	No	5 (80.0)	115 (96.6)		

GA	< 37 weeks	1 (20.0)	23 (19.3)	≤ 0.001	0.970
	37–42 weeks	4 (80.0)	96 (80.7)		

Vaginal exam	Yes > 5	0 (0.0)	19 (16.0)	2.060	0.357
	Yes ≤ 5	1 (20.0)	42 (35.3)		
	No	4 (80.0)	58 (48.7)		

Status of membrane	Ruptured > 12 h	0 (0.0)	12 (10.1)	1.590	0.451
	Ruptured ≤ 12 h	0 (0.0)	17 (14.3)		
	Intact	5 (100.0)	90 (75.6)		

Chorioamnionitis	Yes	0 (0.0)	0 (0.0)	≤ 0.001	≤ 0.001
	No	5 (100.0)	119 (100.0)		

Other infection	Yes	0 (0.0)	0 (0.0)	≤ 0.001	≤ 0.001
	No	5 (100.0)	119 (100.0)		

*Note:* Fisher's exact test.

**Table 8:** Association between SSI following CS and participants' intraoperative and postoperative history.

Variables		SSI	No SSI	Chi-square	*p* value
Type of CS	Elective	3 (60.0)	42 (35.3)	1.267	0.260
	Emergency	2 (40.0)	77 (64.7)		

Indication for surgery	Previous CS	3 (60.0)	50 (42.0)	1.024	0.599
	Malpresentation	0 (0.0)	15 (12.6)		
	Fetal distress	2 (40.0)	54 (45.4)		

Prophylaxis antibiotics	≤ 1 h to surgery	2 (40.0)	72 (60.5)	0.838	0.360
	> 1 h to surgery	3 (60.0)	47 (39.5)		

Type of anesthesia	Spinal	3 (60.0)	107 (89.9)	4.288	0.038
	General	2 (40.0)	12 (10.1)		

Blood transfusion	Yes	0 (0.0)	48 (40.3)	3.291	0.070
	No	5 (100.0)	71 (59.7)		

Skin incision	Pfannenstiel	5 (100.0)	119 (100.0)	≤ 0.001	≤ 0.001
	Midline	0 (0.0)	0 (0.0)		

Procedure duration	≤ 1 h	5 (100.0)	112 (94.1)	0.610	0.435
	> 1 h	0 (0.0)	7 (5.9)		

**Table 9:** Association between SSI following CS and symptoms experienced during the first 30 days (because nonparametric).

Symptom	*N*	Median	IQR	*p* value
Fever				
SSI	5	4.0	4.0	0.040
No SSI	119	1.0	1.0	
Redness				
SSI	5	4	2.0	≤ 0.001
No SSI	119	1.0	1.0	
Edema				
SSI	5	4.0	1.5	≤ 0.001
No SSI	119	1.0	1.0	
Ecchymosis				
SSI	5	2.0	2.0	0.147
No SSI	119	1.0	1.0	
Purulent drainage				
SSI	5	5.0	2.0	≤ 0.001
No SSI	119	1.0	0.0	
Wound closure complications				
SSI	5	5.0	3.50	0.002
No SSI	119	1.0	1.0	

*Note:* Mann–Whitney test was performed, level of significant at *p* < 0.05.

Abbreviation: IQR = interquartile range.

## Data Availability

All relevant data generated or analyzed during this study are included in this manuscript.
